# 3D electron diffraction goes multipolar

**DOI:** 10.1107/S2052252524003774

**Published:** 2024-04-26

**Authors:** R. Beanland

**Affiliations:** a Department of Physics, University of Warwick, Coventry CV4 7AL, United Kingdom

**Keywords:** electron crystallography, 3D electron diffraction, microcrystal electron diffraction, dynamical refinement, transferable aspherical atom model, multipolar scattering factors, quantum crystallography

## Abstract

Over 30 years ago, it was shown that bonding between atoms has a noticeable effect on convergent beam electron diffraction patterns. The paper by Olech *et al.* [(2024). *IUCrJ*, **11**, 309–324] demonstrates that its influence is also clearly present in 3D electron diffraction data, opening up new possibilities for quantum crystallography.

The article by Olech *et al.* (2024 ▸) in this issue of **IUCrJ** marks an important step forwards in accessing some of the wealth of information that can be obtained from materials using electron diffraction (ED). It brings together an accurate description of electron scattering and quantum crystallography models of bonding between atoms in a crystalline solid. The field of ED is developing rapidly at the moment, as quantitative measurements and computer control of electron microscopes [and indeed dedicated electron diffractometers (White *et al.*, 2021 ▸; Merkelbach *et al.*, 2022 ▸)] are beginning to give routine access to high-quality data that has for so long been the norm in X-ray diffraction (XRD) and neutron diffraction (ND). Approaches that have been developed over decades in the latter two fields are now being applied to ED, opening up whole new fields of investigation, particularly for nanoscale crystals that can be easily studied by electrons due to their much stronger interaction with matter.

This strong interaction, inherent in electron diffraction – and resultant multiple scattering – brings many benefits that are beyond the reach of measurements where single scattering dominates. Friedel’s law, mandating equivalence of the intensity of reflections related by inverting the scattering geometry, does not apply, allowing absolute structure to be determined (Buxton *et al.*, 1976 ▸; Brázda *et al.*, 2019 ▸). Additionally, low-order structure factors may be measured much more accurately using ED in comparison with XRD or ND, giving the chemical (bonding) information that is the focus of this article. However, access to the rich and deep information available in ED comes with the cost of increased complexity in the models required to give a good description of the data and the desired measurements.

The benefits, and limitations, of ED have been apparent for some time, (Spence, 1993 ▸) and bonding effects were determined using dynamical scattering theory in combination with convergent beam electron diffraction (CBED) patterns over thirty years ago (Zuo & Spence, 1991 ▸). Nevertheless, the measurement was not straightforward, requiring manual control of both the transmission electron microscope (TEM) and the crystal goniometer to select and orient a suitably defect-free region of a thin crystal for the diffracted beam(s) of interest; recording the pattern on film; digitizing the negative; and refining to a solution using the limited computing resources of the time. The result was a handful of low-order electron structure factors that, in combination with higher-order structure factors determined by XRD, could be used to measure deviations from the neutral, spherical atoms of an independent atom model (IAM) (Zuo *et al.*, 1999 ▸).

Olech *et al.*’s article clearly shows that the success of CBED can be replicated by the various techniques that fall under the acronym 3DED (Gemmi *et al.*, 2019 ▸). Importantly, computer control of both the electron optics and the noise-free detectors of current methods makes them relatively straightforward experimentally. Furthermore, the use of a parallel beam removes the restriction on lattice parameters imposed by CBED, in which the convergence angle must be smaller than the smallest Bragg angle in the diffraction pattern. These advantages mean that 3DED techniques are relevant to materials that are of interest to a wide range of scientists and crystallographers. Here, the material chosen to demonstrate the approach is an organic, beam-sensitive molecular crystal, which yielded only a few reliable measurements even with specimen cooling. That a refinement performed on this less-than-ideal data using an aspherical multipolar atom model not only gave a significantly better match between model and experiment, but yielded chemically sensible results, demonstrates the strong influence of bonding effects in ED and is very encouraging for future studies. Furthermore, the ability to qualify different versions of transferable aspherical atom model (TAAM) against experimental data as demonstrated in this article will undoubtedly drive improvements in quantum crystallography.

This article is yet another demonstration that improvements in technology, technique and modelling in ED are opening avenues of research that previously have been only partially visible. There is yet some distance to travel.
